# An Opto-Electronic Sensor for Detecting Soil Microarthropods and Estimating Their Size in Field Conditions

**DOI:** 10.3390/s17081757

**Published:** 2017-08-01

**Authors:** Csongor I. Gedeon, Norbert Flórián, Péter Liszli, Beáta Hambek-Oláh, Oxána Bánszegi, Judit Schellenberger, Miklós Dombos

**Affiliations:** 1 Institute for Soil Sciences and Agricultural Chemistry, Centre for Agricultural Research, Hungarian Academy of Sciences, Herman Ottó út 15, H-1022 Budapest, Hungary; florian.norbert@agrar.mta.hu (N.F.); schellenberger.judit@agrar.mta.hu (J.S.); dombos.miklos@agrar.mta.hu (M.D.); 2 Deákdelta Ltd. Irinyi János u. 1, H-6727 Szeged, Hungary; liszlipeter@gmail.com; 3 Department of Physical Geography and Geoinformatics, University of Szeged, Egyetem u. 2., 6722 Szeged, Hungary; olah.bea@gmail.com; 4 Instituto de Investigaciones Biomédicas, Universidad Nacional Autónoma de México, AP 70228, CP 04510 Ciudad de México, Mexico; oxana.banszegi@gmail.com

**Keywords:** wireless sensor, monitoring system, automated sensing, soil microarthropods, soil monitoring, soil biodiversity, detection, size estimation, EDAPHOLOG

## Abstract

Methods to estimate density of soil-dwelling arthropods efficiently, accurately and continuously are critical for investigating soil biological activity and evaluating soil management practices. Soil-dwelling arthropods are currently monitored manually. This method is invasive, and time- and labor-consuming. Here we describe an infrared opto-electronic sensor for detection of soil microarthropods in the size range of 0.4–10 mm. The sensor is built in a novel microarthropod trap designed for field conditions. It allows automated, on-line, in situ detection and body length estimation of soil microarthropods. In the opto-electronic sensor the light source is an infrared LED. Two plano-convex optical lenses are placed along the virtual optical axis. One lens on the receiver side is placed between the observation space at 0.5–1 times its focal length from the sensor, and another emitter side lens is placed between the observation space and the light source in the same way. This paper describes the setup and operating mechanism of the sensor and the control unit, and through basic tests it demonstrates its potential in automated detection of soil microarthropods. The sensor may be used for monitoring activities, especially for remote observation activities in soil and insect ecology or pest control.

## 1. Introduction

There is a strong relationship between diversity, abundance of soil organisms and soil health [1,2]. For instance, organic matter decomposition is regulated by a complex food web of soil organisms thus loss of soil biodiversity can lead to degradation of decomposition. One major challenge to measure and monitor abundance and diversity of soil organisms is their strong spatio-temporal heterogeneity [3,4,5]. The fluctuations of soil fauna activity [6,7,8,9] often leads to inefficient experimental designs with low number of temporal or spatial repetitions.

As a result of the strong interaction between physical, chemical and biological properties of soils, soil organisms are considered highly responsive, sensitive indicators of soil quality. Their abundance is the highest in the leaf litter layer and the upper 10–20 cm of the soil [10,11,12]. One of the four main functional and size groups of soil organisms is the mesofauna (0.2–4 mm) [13], whose abundance and diversity are good indicators of biological soil quality and degradation [14,15]. However, their trapping, extraction, and then classification into groups or identification require a lot of work [16].

By and large, to assess the state of soil biodiversity and the risks of its loss there is a need for meaningful, easily understandable, measurable, and policy relevant indicators [17]. The technique should be as non-invasive as it possible. They should also incorporate as many taxa as possible so that the more distinct and differential results can be expected. There are other points to be taken into account, such as long-term continuity and consistency, appropriate spatial and temporal measurement resolution, assessment of reliability by adequate quantification of precision, bias and accuracy, and finally documentation of all potentially relevant boundary conditions by suitable metadata. From a technical aspect, long-term continuity and consistency of monitoring means that we are able to monitor the soil mesofauna for a long time without further disturbance in the soil ecosystem. Concerning accuracy, it means that we should shorten the measurement periodicity and improve measurement quality (precision and bias). This is the only way to detect reliable signals and trends at an early stage and to identify problems in time so that we can decide what measures are needed. From an electromechanical aspect these conditions mean that the monitoring device of soil fauna should work without any modifications for several months and as a result the energy source should be able to provide sufficient power for operation within extreme conditions, such as in heavy rains and then saturated soil.

As a result of the need for meeting those abovementioned needs of soil and insect monitoring, automated detection and counting of arthropods have been developed. These methods are usually based on photoelectric detection. For instance, the Electronic Grain Probe Insect Counter (“EGPIC”) [18] developed to remotely observe insects moving in grains. The device detects the entry and size of objects falling into the detection space illuminated by an infrared light-emitting diode in the trap created in the target area with the help of one or more electrically controlled photodiodes. Then data are shaped and amplified using analog and digital circuits and forwarded to a computer via a parallel port. There they are evaluated and registered or forwarded if necessary [18]. In the improved detection system [19] the arrangement of the control electronics of the photoelectric sensor unit has been modernized by the incorporation of a microcontroller; the accuracy of detection may thereby have been manipulated by adjusting certain parameters of the signal amplifier, and a number of remotely placed photoelectric sensors have been organized into one common data system by enabling these to communicate with a central unit using radio-frequency signals [20]. Further and similar opto-electronic sensors for insect applications, such as insect detection and monitoring, are well detailed in the work of Engel and co-authors or others [21,22,23,24,25]. These examples demonstrate that automated and remote detection of arthropods have received intensive attention because this otherwise manual activity has several limitations. Our detection system uses the same approach and opto-electronic solutions as the abovementioned authors but we have stepped further to develop a less expensive sensor and system that can be integrated into a cylinder-shape, plastic, water and dustproof housing, and then installed into the soil. Compared with other opto-electronic sensors and from a technical viewpoint the environmental challenges underground are different from challenges aboveground: soils are or can be humid or even saturated and its texture varies greatly as a result of different particles and minerals of different sizes, such as sand, silt, and clay particles. The new animal trap, which provides the housing for the electronics, involves the sensor and other electronics, and can guarantee a continuous and reliable operation underground independently from different local, weather and soil conditions. Another important point was to guarantee the least disturbance to the soil ecosystem, which is a dynamic system in which different minerals, dead organic material and live organisms interact and establish a complex abiotic-biotic network. Compared with earlier sensors and systems which used photovoltaic technology to detect flying insects or insects in stored products our novelty was to adapt this technology into the soil ecosystem.

**Figure 1:**
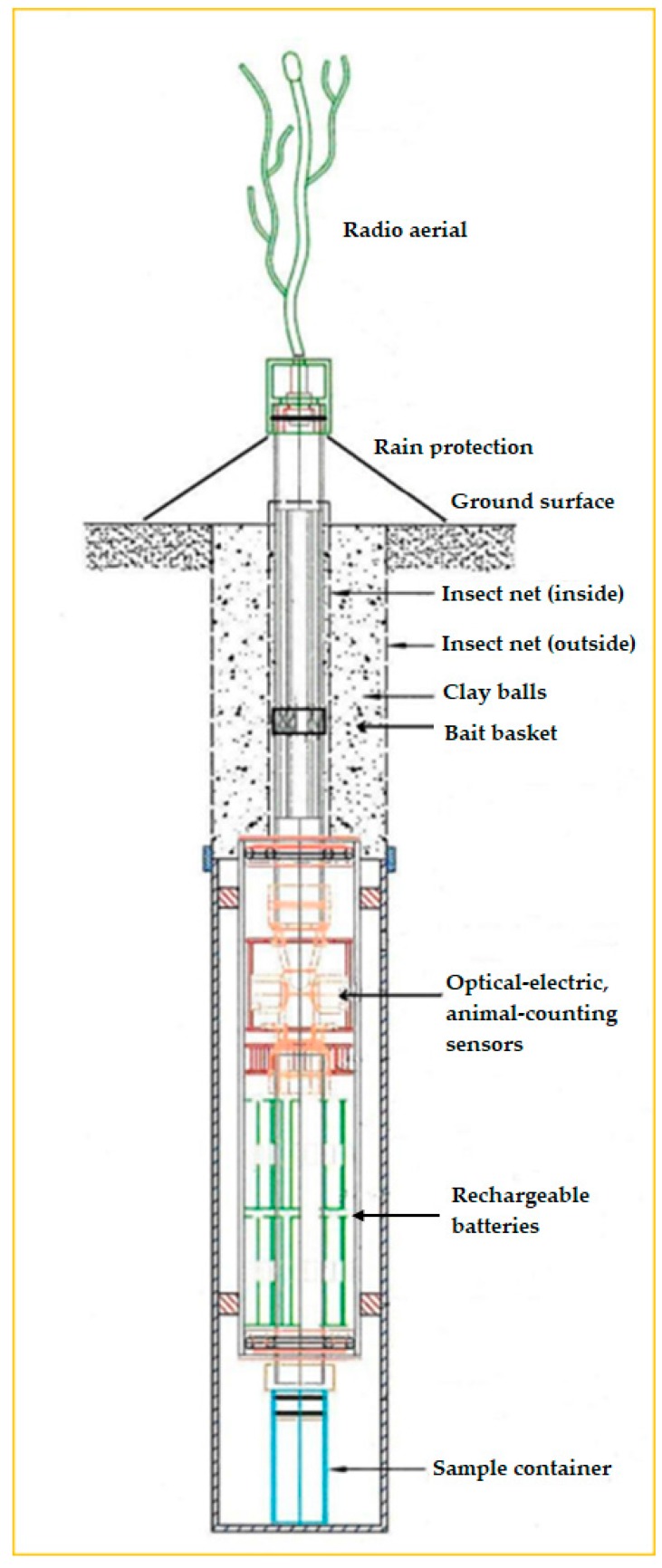
EDAPHOLOG probe provides the house for the opto-electronic sensors. It is part of a new soil environmental monitoring tool, called EDAPHOLOG System. EDAPHOLOG System is constructed for measuring parameters of the soil biota to assess soil biological activity.

We developed a novel soil probe and sensing system for detecting soil microarthropods, which is embedded in a new system called EDAPHOLOG monitoring System [26]. The EDAPHOLOG System is composed of an innovative sampling tool, the probe (Figure 1), designed to spatially and temporally sequential sampling schemes, and a novel detecting system, the opto-electronic sensors, and an own logging system, the microcontroller, for GSM remote data-transmitting and date storage, which is connected temporally and as necessary to the probe by radio signals.

The trapping part of the probe is similar to pitfall traps, which is based upon the fall of soil arthropods into the trap. Consequently the probe is sunk into the soil in such a way that the upper part of EDAPHOLOG probe is to be located near surface, at a depth of about 15–20 cm. As a result soil-dwelling (euedaphic) microarthropods can enter into the probe and then are captured and stored in the sample container. On-line monitoring is aided by automatic data transfer and probe control using radio and GPRS communication systems. The result of our development and most important part of this system has been a sensor with optimized light transmission, and relatively cheap and simplified electronics, which uses significantly less energy than conventional devices, and provides relatively precise counting data.

The primary objective of this paper is to describe in detail the opto-electronic sensor developed to operate in the soil environment to detect microarthropods and estimate their body length. The technical review includes the determination of detection threshold and efficiency, minimization of signal noise, and demonstration of characteristic signal patterns in response to animate and inanimate objects with different lengths (sizes). We also briefly introduce the components of the prototype of an optional, cylinder shape housing for the sensor but focus on the sensing unit and its basic detecting function. This housing serves as a microarthropod trap (EDAPHOLOG probe [26]) to catch soil arthropods (Figure 1). Our further objective, while introducing the sensing unit and the trap, is to demonstrate its potential for the assessment of numerical abundance of soil mesofauna microarthropods, as an alternative to current, manual techniques. These techniques combine pitfall traps and extraction of soil fauna from soil samples in Tullgren or MacFadyen apparatuses [27,28,29]. Sample processing and analyzing are extremely time- and labor-intensive with both apparatuses.

## 2. Materials and Methods

### 2.1. Operation of the Sensor

The device is based on an opto-electronic sensing photodiode (hereafter as sensor) for the detection of small objects—preferably falling insects in the size range of 0.4–10 mm. Analogue sensor circuitry detects the falling particles. The light source is infrared light-emitting diodes, and multiple plano-convex optical lenses have been placed along the virtual optical axis connecting the light-emitting diode and the sensor. On the emitter and receiver sides lenses were placed between the observation space and the light-emitting diode or sensor. Lens was always placed at 0.5–1 times of its focal length from the light-emitting diode or sensor.

**Figure 2:**
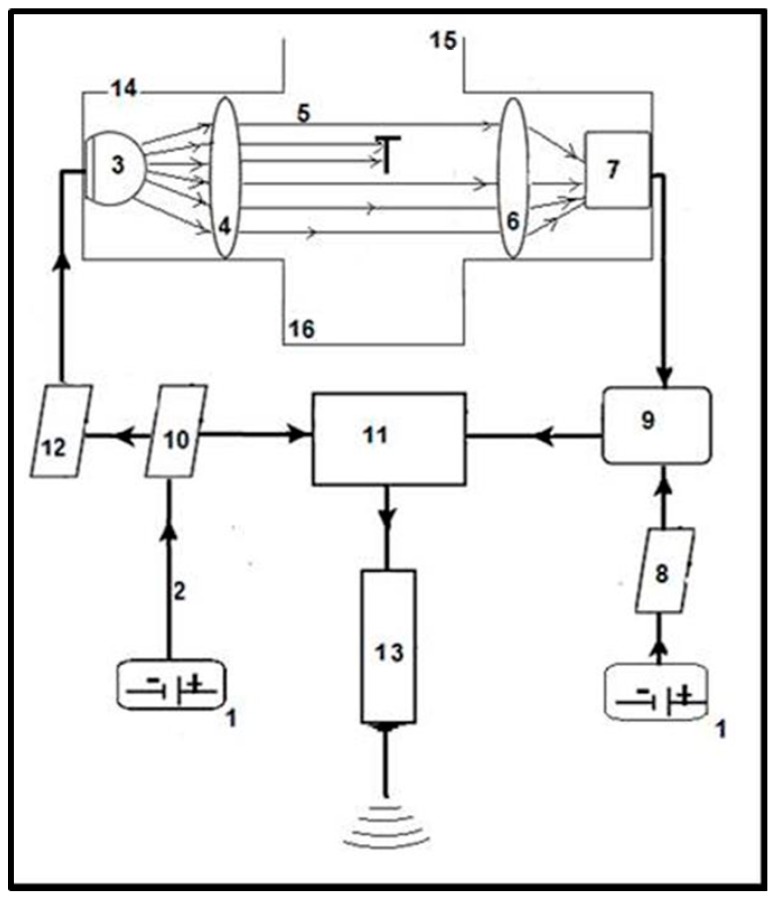
Operational flow chart of detecting and measuring falling-in objects through the dual optical-electronic sensors. The sensor has an electronically controlled light-emitting diode (**3**) containing the observation space (**5**), fitted to the housing (**14**) and a sensor (**7**) connected through the signal amplification module (**9**) to the electronic control unit (**11**). The power supply of the control unit (**11**) is supplied from the power source (**1**) through the supply circuit (**10**) from where, by the insertion of a filter (**12**), the power supply of the infrared light-emitting diode (**3**) is also provided. The control unit (**11**) has a communication module (**13**) containing the radio transmitter. The elements of the electronic arrangement are connected to each other via cables (**2**). The sensor (**7**) is a photodiode in photovoltaic operational mode and it is connected to a power supply unit (**8**) and multistage amplifier module (**9**). Two plano-convex lenses are placed along the virtual optical axis connecting the light-emitting-diode (**3**) and the sensor (**7**) passing through the observation space (**5**), with an emitter side lens (**4**) placed between the light source (**3**) and the observation space (**5**) at 0.5–1 times of the focal length from the light-emitting-diode (**3**), and a receiver side lens (**6**) placed between the observation space (**5**) and the sensor (**7**) at 0.5–1 times the focal length from the sensor (**7**). For a particular embodiment, the power sources (**1**) are preferably 3 V batteries, the infrared light source (**3**) is a TSAL6200 type light-emitting-diode with a wavelength of 940 nm, the casing of the sensor photodiode (**7**) has a minimal attenuation for light around 940 nm and the distance between the light-emitting-diode (**3**) and the sensor (**7**) is 30–60 mm. The optical lenses fitted (using screws enabling fine distance adjustments) to the housing (**14**) containing the observation space (**5**) and supporting the light source (**3**) have diameters of 13 mm, thicknesses at the centre of 3.6 mm and 2.4 mm at the edge with focal lengths of 20 mm. The distance between the lenses is preferably 25–40 mm, and the distance between the sensor (**7**) side and receiver (**6**) side lenses is about 0.5–1 times the focal length. If the light source (**7**) was infrared laser, the emitter side lens (**6**) would not be necessary, but the cost of the laser and the energy demand of the operation and the cost of the control elements would also be higher. (**15**) Entry point of microarthropods into the sensor and observation space. (**16**) Exit point of microarthropods from the sensor.

The principle of the operation is that light intensity emitted by the light-emitting diode and then perceived by the sensor changes as soon as a falling object crosses the observation space. One of our priorities was to be able to detect small objects (≥0.1 mm) therefore we amplified the standby noise signal to around the threshold level of detection. Since standby noise signal follows Gaussian distribution consequently the chance of false detection always remains. The sensor is used in the photovoltaic mode because it can maintain a minimal leakage current when the incoming infrared light develops bias voltage across the terminals of the photodiode. This voltage is proportional to the intensity of the incoming light, which is also proportional to the size of the falling-in object. When an object falls into the observation space it triggers detection and size estimation (Figure 2). Then the signal is analyzed by a microcontroller, which will determine to keep or ignore detection triggered by the object on the basis of the signal’s voltage amplitude.

Because the whole equipment runs on batteries and the whole system is developed to work on battery power for several months, from the point of view of electro-technical construction the requirement was to use the least possible current consumption. As low as about 0.5 mA current is sufficient to drive the emitting light-emitting-diode to get acceptable sensitivity and signal to standby noise signal ratio.

### 2.2. Signal Amplifier

**Figure 3:**
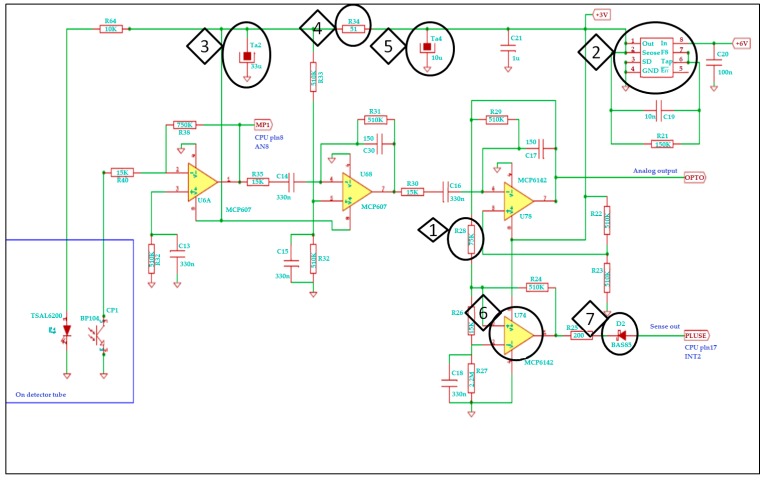
Signal amplifier circuit schematics. Essential parts of the circuit are circled and numbered (◊), and their functional role is explained hereafter. (**1**) R28 (R = 75 KΩ), hysteresis comparator: Detection threshold is primarily set up here at the last hysteresis comparator of the amplification chain. (**2**) U5, voltage stabilizer integrated circuit: It provides independent supply voltage for the analogue signal amplifier to decrease electric noise of the signal amplifier. (**3**–**5**) Parts Ta2, R34, Ta4: Reduce electric noise. (**6**) U7A, operational amplifier comparator: Sensing is initialized by a negative impulse from this comparator via diode D2 (**7**) to the CPU. It results in higher system clock frequency of the CPU, which then starts detection.

The signal amplifier consists of 3 stages to achieve high enough amplitude to detect small invertebrates (Figure 3). The first stage functions as an impedance matching circuit and has direct current coupling. The further stages are coupled through capacitors to cut off direct current instability and amplify only alterations caused by falling-in objects. The time constant is 30 ms between signal amplifier stages. On the one hand, this period of time is long enough for a falling-in object to go across the observer space in the sensor. On the other hand, environmental processes that may increase standby noise signal cannot amplify the signal within this short period of time. The final stage is a hysteresis comparator. The value of the hysteresis is set to about 80 mV. This way, as much as 80 mV signal can already trigger the sensing process. Despite the fact that both the signal amplifier and the CPU unit run from 3 V power supply, we still cannot use a common power supply as the amplifier must run from a very low noise power supply.

### 2.3. CPU Unit

The CPU unit controls the operation (detection and size estimation) of the whole EDAPHOLOG probe including the light-emitting diode and the sensor. The CPU is a Microchip PIC18F14K22 microcontroller. In idle state it runs at a 31 KHz clock rate to save power. When the analogue circuitry detects an object in the sensing field, it generates a trigger pulse (negative impulse) that awakes the CPU and the firmware switches to a higher system clock of 2 MHz. The A/D converter starts at this point and firmware accumulates the deviations from the baseline. The accumulated value is proportional to the size of the fallen object. After each sensation the system transmits the recorded event through a 433 MHz RF module to a central communicator. This GSM/GPRS communicator logs each capture from several opto-electronic sensor units. Once a day this module transmits the recorded data to a central server.

### 2.4. Laboratory Testing of Detection Efficiency

We detected and counted spherical sample pieces (between 0.3 and 2.3 mm) and two types of live soil microarthropods (between 1.6 and 7.5 mm) with known size by dropping them manually into the optical-electronic sensors. We grouped probe pieces into size categories of 100 µm each: from 0.3 to 2.0 mm there were 18 size categories, and 2.2 and 2.3 mm. Animals were also grouped into size categories: 1.6, 2.1, 3.2 and 7.5 mm. Number of samples for each size category was between 20 and 70 (details in figures). All these categories did not overlap with each other so detection success of a size group reliably represented the size category. We used spherical (inanimate) and elongated (animate) objects to compare if elongated shape influences detection success significantly. Springtails, *Folsomia candida* (white bodied, hairless species with short antennae and furca) and *Orchesella cincta* (strongly pigmented, hairy species with long antennae and furca), were used for these tests. These animals were obtained from standard breeds from our laboratory or captured from our research area and kept in laboratory for several weeks before the experiment. The body length of each specimen was measured to the nearest 0.001 mm following the methods by Bánszegi and co-authors [30]. The method consists of three elements: (1) an imaging device (CollScope); (2) photographing software; (3) an ImageJ macro for image processing, measurement and data analysis. Body length of live microarthropods can be automatically measured by this method. By these measurements detection success of the sensor and its measurement range (minimum threshold) was tested.

### 2.5. Signal Patterning during Detection and Size Estimation of Falling-In Objects

In addition to detection, signals emitted by the photo-sensor circuits in response to objects falling into the observation space contain information about the size of the detected objects. We tested if soil microarthropods with different body lengths generate significantly different output signals.

### 2.6. Statistical Analysis

In the statistical analysis we primarily wanted to estimate success rate (%) and minimum threshold level of detection of falling-in objects. For determining detection success of falling-in objects with different sizes, we applied exact binomial tests for given probabilities. In exact binomial test we tested whether the experienced proportion of successful detection of falling-in objects was significantly greater from a population with a given success of detection of falling-in objects. The advantage of the exact binomial test compared with chi square test for given probabilities was that we could test if detection success was greater than a given probability (“one-sided hypothesis”) and it can be applied for small and large samples. Falling-in, inanimate objects were isometric (spherical) consequently their detection primarily depended on their diameter, which corresponded to the minimum threshold level and measurement range of detectability of the sensor. Statistical analyses were carried out using R commander [31].

## 3. Results

### 3.1. Baseline, Noise and Detection Threshold

**Figure 4:**
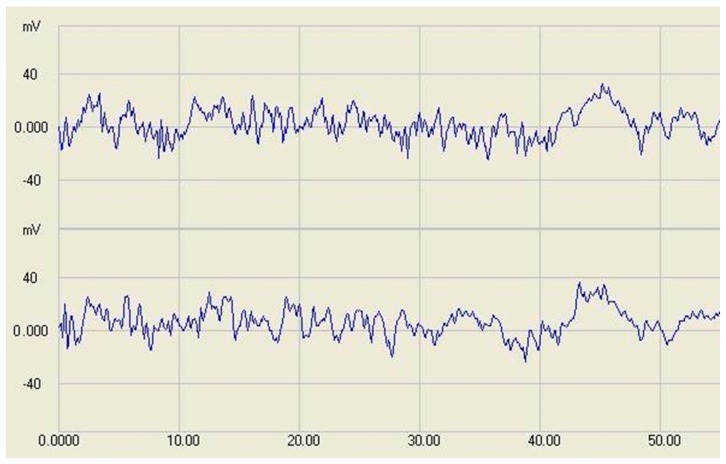
Baseline and standby noise signal of the sensor (no-detection state) at two sensors. The X-axis shows time in ms and Y-axis shows voltage in mV.

To maintain low current consumption, the least possible infrared (IR) light is applied. Therefore the photodiode works at a very low current and has high impedance. This accounts for the high noise amplitude and the sensitivity against external noise sources. The comparator has about 70 mV hysteresis and 17 mV bias offset to maintain high-level output in idle state. Figure 4 shows the sensor signals at no-detection state, which provides the baseline and the noise of the signaling. The general noise amplitude with all circuits was about 60–70 mV from peak to peak.

We tested ten sensors with different current levels to find the optimal sensing threshold level. Three current levels were chosen in the final test—70 mV, 82 mV and 100 mV—and were evaluated on the whole set of prototype circuits. These values represented 1.75-, 2- or 2.5-fold threshold levels respectively. Then we examined how much noise is generated on the sensor with the three different current levels. 10 sensors were in operation throughout 24 h and then the number of false detections registered by the sensors was counted. The upper tube of the sensor was covered by a transparent foil to prevent any particle from falling into the sensing field during the test. In Table 1 the number of false detections is shown in every two h. It can be seen that with the 70 and 82 mV levels, there are too many false detections, but with the 100 mV setting not a single detection occurred within the 24-hour-long test periods.

### 3.2. Current Consumption

Since the system is to work on battery power source for several months, the least possible current consumption was a crucial factor in developing the analogue sensor circuits, the optical sensors and the microcontroller. The EDAPHOLOG probe containing the sensors can operate reliably for 2–3 months at which point it can be recharged with a contact-free inductive energy charging system. The advantage of the wireless charging is that the probe containing the sensors and electronics does not have to be dismantled during recharging; consequently, a closed water-, and dustproof housing can be built around them.

### 3.3. Efficiency and Minimum Threshold Level of Detection

**Figure 5:**
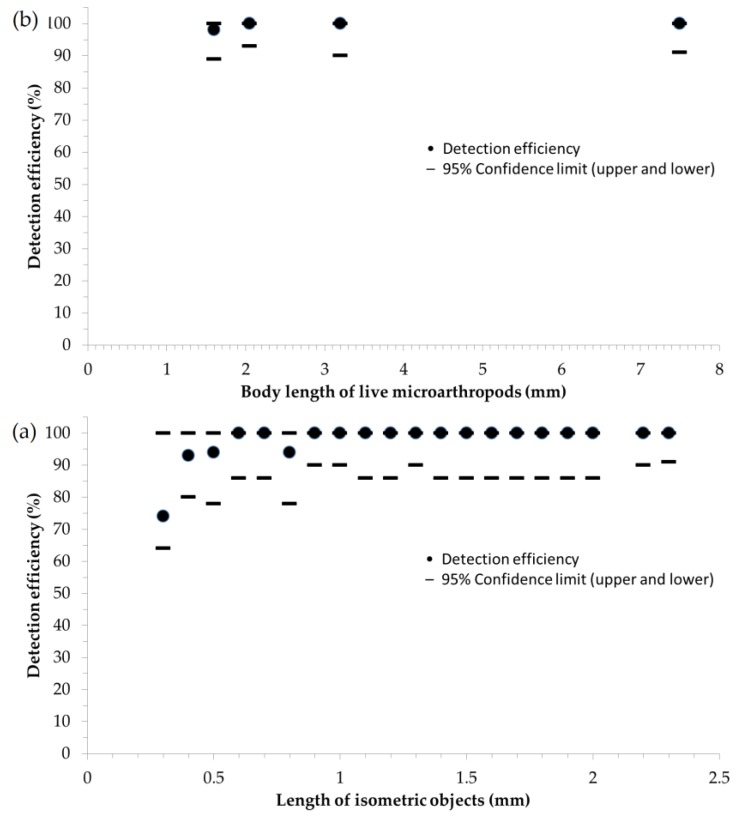
(**a**) Detection efficiency of isometric granules in twenty, distinct size categories (the number of samples are between 20–70 in these size categories). (**b**) Detection efficiency of soil microarthropods in four, distinct size categories (the number of samples are between 30–44 in these size categories). Dash lines show the lower and upper 95% confidence limits of detection efficiency.

Detection success of objects with a diameter of 0.3 mm was 74%. We have found that detection efficincy remained above 90% along all size categories from 0.4 mm, and even reached 100% above 0.9 mm. The size range between the smallest and largest objects corresponds to the minimum threshold level (0.3 mm) and measurement range of the sensor (0.3–7.5 mm). The overlap between the 95% confidence intervals for the probaility of successful detection of artificial (spherical) objects (Figure 5a) and live (elongated) springtails (Figure 5b) shows that there is no siginficant difference in the detection of falling-in objects between live and artificial objects.

### 3.4. Signal Patterning during Detection and Size Estimation of Falling-In Objects

**Figure 6:**
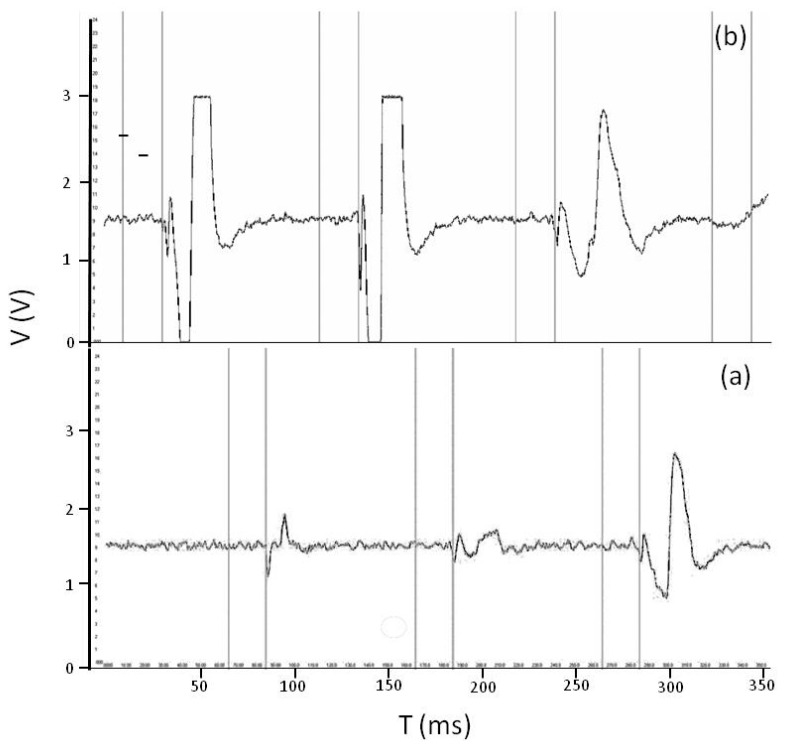
Figures show the electrical signals generated by three smaller Collembola (Folsomia candida) (**a**) and three larger Collembola (Orchesella cincta) (**b**). The first vertical line designates when the animal fell in and detection started. The signal was saved between second and third vertical lines. X- axis shows time period in ms and Y-axis shows voltage in mV.

To evaluate the ability and accuracy of insect detection, records of the analogue signals were made on two Collembola species. Figure 6 shows three smaller (*Folsomia candida*; Figure 6a) and three larger Collembola individuals (*Orchesella cincta*; Figure 6b) while they are falling into the observation space and generate signals. Larger animals generated electrical signals with larger amplitude (Figure 6b) and longer interrupted light periods (Figure 6b) compared with smaller animals (Figure 6a).

**Figure 7:**
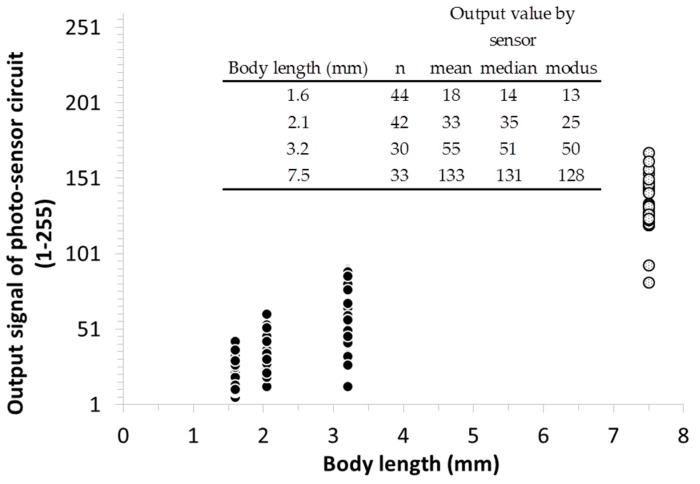
It shows the output signal converted into a dimensionless numerical output value between 1 and 255. Folsomia candida is marked with black circles and Orchesella cincta with white circles. The magnitude of the output value corresponds to the body lengths of the animals.

The output signals were plotted against measured body sizes in Figure 7. The larger species (Orchesella cincta, 7.5 mm) showed a significantly higher output signalling compared to the smaller species (Folsomia candida, 1.6 mm, 2.1 mm, 3.2 mm). Summary statistics of the output values shows that the distribution of signal output points are close to normal. Consequently it indicates that the distribution of the output data for each body length categories may be used for estimating body lengths of soil microarthropods in these size ranges.

Based on electronical tests conducted on soil microarthropods with different sizes the sensing algorithm has been developed so that the output signal, which is millivolt, can be converted into numerical output value (between 1 and 255). The numerical output value does not change linearly but yet able to detect and differentiate distinct sizes of the sensing target (see details in [26]). Detecting and size estimating of falling-in objects reaching the observation space is initiated by a comparator in case the optical signal exceeds the threshold level.

## 4. Discussion

The main importance of the currently described sensor is the usage of photoelectric technology to detect soil arthropods in the field, real time, and with minimal disturbance to the soil ecosystem. Those examples we mentioned in the introduction, such as the EGPIC [18,19] and other sensors based on photodiodes [21,22,23,24,25], had applied this technology for in situ pest control and integrated pest management of flying or above ground insects. Numerous technological innovations have been recently developed for the automated monitoring of arthropods or agricultural pests in particular [32,33,34]. Soil microarthropods are integral part of the soil ecosystem and they deliver several ecosystem functions and services. The automated detection and monitoring of their activity-density [35] are therefore an important need of (agro-)ecology. We introduced a low-cost sensing system to detect soil microarthropods that can be built into a field probe, such as the EDAPHOLOG probe [26]. The field-proof (i.e. water-resistant) housing for the sensor can guarantee long-period field application. As a result of the low energy consumption of the electronic parts it can operate for 2–3 months without recharging. Moreover, the in-built sensor can reduce monitoring effort dramatically, and can provide better temporal and spatial resolution. Its low power consumption and as a result long-life battery requires almost no maintenance and provides real time data for end users about the activity-density of soil microarthropods.

Our results showed that in the size range of 0.4 and 7.5 mm the sensor gave reliable, nearly 100%, detection efficiency of falling-in objects. These results on detection efficiency are in accordance with the results of Dombos and co-authors [26], where the EDAPHOLOG system including the opto-electronic sensor was tested in both field and laboratory conditions on live animals. Our results on accuracy of detection were in accordance with that study: detection efficiency of specific size groups in our study fell in the confidence interval of the statistical model applied in Dombos and co-authors [26]. However, we must keep in mind that detection efficiency dropped to 74% at 0.3 mm due to the 100 mV minimum threshold level. Although this higher, minimum threshold level was necessary to decrease the sensor’s over-sensitivity and as a result avoid false detections, it indicates that the sensor’s detection efficiency declined rather sharply towards the smaller size groups (≤0.3 mm). Based on practical experience, increasing the sensitivity of a sensor unit solely composed of photoelectric sensors produces a high number of false signals, while setting the sensitivity lower will result in the uncertain detection of falling objects with 1–2 mm in size. For purposes of insect ecology, soil science, and plant protection, a detector with a higher level of protection against damage and more refined sensitivity with a preferably lower energy demand are more advantageous. The uncertainty of the detection of small animals (≤0.3 mm) can potentially lead to underestimating small microarthropods. However, according to a comprehensive study of Schon and co-authors [36] on Oribatida abundance in pastures at the depth between 0–15 cm, approximately 80% of specimens were found to be in the medium and large body-size classes (medium size: 0.3–0.75 mm; large size: >0.75 mm). Mercer and co-authors [37] looked at body length distribution of adult Acari, Collembola, and other insects on the Marion Island, Republic of South Africa, and found it to be skewed above 0.6 mm. Tamura (1974) studied [38] populations of a single Collembolan species, *Tomocerus varius*. Although its body length ranged between 0.04 and 1.65 mm, at around 0.7 mm there was a large depression in the frequency of specimens and the majority of animals in the samples were larger than 0.7 mm. These and other studies [39] suggest that the opto-electronic sensor can reliably detect and as a result estimate the abundance of soil microarthropods in the near surface between 0–20 cm. Nevertheless, should very small individuals dominate the soil microarthropods community, the abundance estimate can be corrected by examining a representative sample of microarthropods in the study site. Then one can establish a ratio of small (≤0.3 mm) animals, and use this number as a correction factor in the estimation of their contribution to the total abundance. Another important finding was that detection efficiency did not differ between elongated microarthropods and spherical objects. The lack of difference between these two groups showed that within the currently studied size measurement ranges material, shape, and weight of falling-in objects or animals (morphotype in Dombos et al. [26]) did not modify detection efficiency significantly.

There have been several technical solutions and we think that there will be more and more in the closer future to improve the automated detection and monitoring of arthropods in different media [33], such as air, soil or stored agricultural products. An important issue that will probably require development in these systems is to solve both detection and accurate size estimation of small invertebrates with low energy consumption. Imaging technologies may cope with the accuracy and precision of automated measurements of small arthropods (individuals) but their relatively large energy consumption remains a challenge. As we have pointed out earlier it is important to avoid the disturbance of the soil ecosystem while one collect data for monitoring purposes. Otherwise data coming from a disturbed ecosystem cannot represent the status and change of that dynamic abiotic-biotic system reliably, and consequently it can be used for management or scientific purposes with limitations. Frequent maintenance of devices, such as collection of trapped specimens or recharging of batteries, would require disturbance of the soil ecosystem therefore their frequency should be decreased to minimum. As a result of these difficulties we cannot suggest a better way for detection of soil arthropods and estimation of their size or activity-density at the moment despite the obvious limitations that we have shown.

Size estimation results seems promising but obviously the system has to be tested more in the field for a reliable evaluation of this function of the sensor. Such work has been already done and ongoing further [26]. The parameters of relationship between the sensor’s output value and the corresponding length may vary because different microarthropods (probably rather larger ones) may enter the observation field of the sensor with different angles of incidence. This phenomenon is probably the result of their distinct shape and air resistance or the active movements of their body parts, such as springtails’ furca [26]. In addition, it is likely that different soil conditions and climatic conditions, such as soil moisture content, will yield different detection efficiencies, because the soil community structure and activity will vary. For instance epigeic springtails are better dispersers than edaphic species, and certain groups of mites and springtails disperse with different speed [40,41]. Dispersal capacity also depends on habitat characteristics [42], such as water content in the soil, soil texture and structure (particle size and distribution), porosity. These will influence sampling efficiency of soil fauna [40,43].

Therefore, the sampling efficiency in field conditions can vary. However, the sensor’s performance can maintain its efficiency on the bases of our test results, and similarly to other field device deployments, it is strongly encouraged to calibrate the monitoring with traps or probes including this sensor to local environmental constraints and conditions before starting a long term monitoring.

## 5. Conclusions

We introduced an opto-electronic sensor with the primary aim of its potential to automated, real-time and in situ monitoring of the abundance and activity of soil microarthropods. The sensor system and supporting electronics integrated in a sealed EDAPHOLOG soil probe [26] can provide either farmers or scientists with a tool to estimate the activity-density or biomass of soil microarthropods. Being an automated system it provides a non-invasive method to count soil microarthropods. Non-invasiveness is a valuable quality of long-period in situ monitoring of soil microarthropods since the soil ecosystem is hidden from our eyes and as a result its functions or characteristics, be they physical, chemical or biological, are usually disturbed during measurements.

## Figures and Tables

**Table 1:** Average number of false detections at three sensing threshold levels (70, 82 and 100 mV) during 24 h.

Threshold Level (mV)	Average Number of False Detections per Two Hours during 24 h											
	0–2 h	2–4 h	4–6 h	6–8 h	8–10 h	10–12 h	12–14 h	14–16 h	16–18 h	18–20 h	20–22 h	22–24 h
70	20	17	22	18	24	19	16	23	22	17	20	24
82	4	6	2	5	3	7	5	6	4	5	2	3
100	0	0	0	0	0	0	0	0	0	0	0	0
